# Functional implications of paralog genes in polyglutamine spinocerebellar ataxias

**DOI:** 10.1007/s00439-023-02607-4

**Published:** 2023-10-16

**Authors:** Daniela Felício, Tanguy Rubat du Mérac, António Amorim, Sandra Martins

**Affiliations:** 1 Instituto de Investigação e Inovação em Saúde (i3S), 4200-135 Porto, Portugal; 2 Institute of Molecular Pathology and Immunology of the University of Porto (IPATIMUP), 4200-135 Porto, Portugal; 3 Instituto Ciências Biomédicas Abel Salazar (ICBAS), Universidade do Porto, 4050-313 Porto, Portugal; 4 Faculty of Science, University of Amsterdam, 1098 XH Amsterdam, The Netherlands; 5 Department of Biology, Faculty of Sciences, University of Porto, 4169-007 Porto, Portugal

## Abstract

Polyglutamine (polyQ) spinocerebellar ataxias (SCAs) comprise a group of autosomal dominant neurodegenerative disorders caused by (CAG/CAA)_n_ expansions. The elongated stretches of adjacent glutamines alter the conformation of the native proteins inducing neurotoxicity, and subsequent motor and neurological symptoms. Although the etiology and neuropathology of most polyQ SCAs have been extensively studied, only a limited selection of therapies is available. Previous studies on SCA1 demonstrated that *ATXN1L*, a human duplicated gene of the disease-associated *ATXN1*, alleviated neuropathology in mice models. Other SCA-associated genes have paralogs (*i.e.*, copies at different chromosomal locations derived from duplication of the parental gene), but their functional relevance and potential role in disease pathogenesis remain unexplored. Here, we review the protein homology, expression pattern, and molecular functions of paralogs in seven polyQ dominant ataxias—SCA1, SCA2, MJD/SCA3, SCA6, SCA7, SCA17, and DRPLA. Besides *ATXN1L*, we highlight *ATXN2L*, *ATXN3L*, *CACNA1B*, *ATXN7L1*, *ATXN7L2*, *TBPL2*, and *RERE* as promising functional candidates to play a role in the neuropathology of the respective SCA, along with the parental gene. Although most of these duplicates lack the (CAG/CAA)_n_ region, if functionally redundant, they may compensate for a partial loss-of-function or dysfunction of the wild-type genes in SCAs. We aim to draw attention to the hypothesis that paralogs of disease-associated genes may underlie the complex neuropathology of dominant ataxias and potentiate new therapeutic strategies.

**Supplementary Information:**

The online version contains supplementary material available at 10.1007/s00439-023-02607-4.

## Microsatellite repeats in dominant spinocerebellar ataxias

Short tandem repeats (STRs or microsatellites) consist of short sequence motifs (1–6 bp) contiguously repeated at a given *locus*, estimated to constitute at least 3% of the human genome (Shortt et al. 2020). STRs are intrinsically unstable and more prone to mutations than other parts of the genome (Ellegren 2000), occurring mostly alterations in the number of repeated units (contractions or expansions). These alterations can happen via slipped-strand mispairing during DNA replication, recombination events (unequal crossing over or gene conversion), or incorrect repair of DNA strand-breakage (Gemayel et al. 2010). STRs occur both in coding and noncoding regions of the genome but those that fall within coding sequences are limited to trinucleotide repeats, often CAG or GCN encoding for polyglutamine (polyQ) and polyalanine (polyA) amino acid tracts, respectively. On the other hand, repetitive *loci* in the noncoding genome may encompass regulatory elements (Sawaya et al. 2013; Fotsing et al. 2019) and function as expression regulators through the modulation of DNA methylation, alternative splicing, and transcription factor binding (Gemayel et al. 2010). In both coding and noncoding regions, large expansions (typically more modest in length in the first) have occurred at some *loci* over human evolution. These expansions, after reaching a given *locus*-specific threshold, trigger cytotoxicity through diverse mechanisms leading to disease (Hannan 2018; Depienne and Mandel 2021).

To date, more than 40 repeat expansion disorders have been discovered to primarily affect the nervous system, including 13 spinocerebellar ataxias (SCAs). In this group of disorders, the length and nucleotide composition of the STR motif differs according to the causative gene: SCA1, SCA2, Machado-Joseph disease (MJD)/SCA3, SCA6-8, SCA10, SCA12, SCA17, SCA31, SCA36, SCA37, dentatorubral-pallidoluysian atrophy (DRPLA), and the recently identified SCA27B (Klockgether et al. 2019; Pellerin et al. 2023). From these repeat-associated SCAs, seven are caused by (CAG)_n_ tracts, which together with Huntington’s disease and spinal and bulbar muscular atrophy make a total of nine polyQ-associated diseases (Hannan 2018; Paulson 2018). Genes involved in polyQ SCAs are ataxin 1 (*ATXN1*; SCA1), ataxin 2 (*ATXN2*; SCA2), ataxin 3 (*ATXN3*; MJD/SCA3), calcium voltage-gated channel subunit α1A (*CACNA1A*; SCA6), ataxin 7 (*ATXN7*; SCA7), TATA-box binding protein (*TBP*; SCA17) and atrophin 1 (*ATN1*; DRPLA) (Table 1). Additionally, SCA12 is also a CAG disorder but the expanded repeat is located in the untranslated region of the gene, typically not encoding polyQ tract proteins (Paulson 2018). In the case of SCA8, the bidirectional transcription of CTG*CAG repeat expanded transcripts in two overlapping genes, ataxin 8 opposite strand and ataxin 8 (*ATXN8OS*/*ATXN8*), produces a CTG-expanded antisense non-coding RNA and a pathogenic polyQ protein (Moseley et al. 2006). Moreover, an alternative repeat-associated non-AUG translation may contribute to the pathogenesis of SCA8 (Zu et al. 2011). Thereafter, SCA2 and SCA7 have been found to present bidirectional transcription, along with Huntington’s disease. The *ATXN2* (CAG)_n_ was found to be bidirectionally transcribed into an antisense (CUG)_n_ (*ATXN2-AS*) transcript in both unaffected and SCA2 affected brain tissues. However, both normal and expanded *ATXN2-AS* RNAs do not seem to be translated by non-AUG translation (Li et al. 2016a). In *ATXN7*, one alternative promoter (intron 5′ to exon 3) has been found to transcribe the spinocerebellar ataxia 7 antisense noncoding transcript 1 (*SCAANT1*). Notably, the *SCAANT1* and *ATXN7* have a synergist transcriptional regulation, which is dysregulated by polyQ expansions (Sopher et al. 2011). Very recently, a novel subtype of SCA caused by a (CAG)_n_ expansions in the THAP domain containing 11 gene has been described in two families (Tan et al. 2023).

**Table 1:** Summarized description of polyQ SCA-causative genes, protein molecular function and main interactors

Disease	SCA1	SCA2	MJD/SCA3	SCA6	SCA7	SCA17	DRPLA
Gene	*ATXN1*	*ATXN2*	*ATXN3*	*CACNA1A*	*ATXN7*	*TBP*	*ATN1*
Chromosome location	6p22.3	12q24.12	14q32.12	19p13.13	3p14.1	6q27	12p13.31
Repeat location	Exon 8	Exon 1	Exon 10	Exon 47	Exon 1	Exon 3	Exon 5
Protein molecular function	Transcription cofactor (regulates RNA transcription and processing)	RNA binding protein implicated in mRNA translation	Deubiquitinase involved in the ubiquitin–proteasome system and transcriptional regulation	Voltage-gated calcium channel subunit (Ca_v_2.1)/transcription factor (ɑ1ACT)	Component of SAGA acetyltransferase complex (transcription coactivator)	General transcription factor from the TFIID complex	Transcription cofactor (recruits DNA-binding proteins to repress transcription)
Interactors (in vitro and/or in vivo)	ATXN1, ATXN1L (BOAT), CIC, HDAC3/4, MEF2, ANP32A (LANP), PQBP1, RBM17, RORA/KAT5 (TIP60), SMRT/NCoR2, RBPJ (CBF1), RFX1, ZBTB5, ZKSCAN1, USP7, UBQLN4 (A1U), COIL, GAPDH, YWHAE (14–3-3 protein)	ATXN2L, PABP, RBFOX1 (A2BP1), DDX6, TDP-43, STAU1, endophilins (SH3GL2/SH3GL3)/CBL/CIN85, RAD23A/B (HR23A/B), VCP, UBR2, α-tubulin, caspase-7, PARK2	CBP/PCAF/P300, HDAC3/SMRT, FOXO4, TBP, PML, beclin-1	CABP1 and SNARE proteins (STX1A and SNAP-25; for Ca_v_2.1 channel), AT-rich and CA-rich DNA elements (for ɑ1ACT)	SAGA proteins (such as ATXN7L3, GCN5, TRRAP, TAF10), CRX, SIRT1, RORA, TBP, HDAC3, α-tubulin, SORBS1 (SH3P12), PSMC1 (S4)	DNA elements, TFIIB, TAFs (TAF1A, TAF1B, TAF1C), NCOA6 (ASC2), DRAP1 (NC2A), DR1 (NC2B), ELF3 (ESX), SPIB, SNAPC1, SNAPC2, SNAPC4, UTF1, BRF2 (BRFU), UBTF (UBF), HSF1, XBP1, MYOD, SP1, NF-γ, PAX5	RERE, HDAC1/2, CBP/TAF4, TBP, MTG8, BAIAP2 (IRSp53), WW domain containing proteins (WWP1, WWP2, WWP3), DVL1, NR2E1 (TLX), FAT1, ITCH, beclin-1
References	Koshy et al. (1996); Matilla et al. (1997); Davidson et al. (2000); Okazawa et al. (2002); Hong et al. (2002, 2003); Chen et al. (2003); Tsai et al. (2004); Mizutani et al. (2005); Serra et al. (2006); Lam et al. (2006); Bolger et al. (2007); Lim et al. (2008); Tong et al. (2011); Gehrking et al. (2011); Suter et al. (2013); Venkatraman et al. (2014); Kim et al. (2014); Coffin et al. (2023)	Shibata et al. (2000); Figueroa and Pulst (2003); Nicastro et al. (2005); Satterfield and Pallanck (2006); Nonhoff et al. (2007); Nonis et al. (2008); Elden et al. (2010); Kaehler et al. (2012); Paul et al. (2018; Weishäupl et al. (2019)	Chai et al. (2001); Li et al. (2002); Evert et al. (2006); Araujo et al. (2011); Ashkenazi et al. (2017); Feng et al. (2018)	Atlas et al. (2001); Lee et al. (2002); Cohen-Kutner et al. (2010); Du et al. (2013)	La Spada et al. (2001); Lebre et al. (2001); Matilla et al. (2001); Chen et al. (2004); Helmlinger et al. (2004); Ström et al. (2005); Zhao et al. (2008); Köhler et al. (2010; Nakamura et al. (2012); Duncan et al. (2013); Stoyas et al. (2017); Papai et al. 2020)	Ha et al. (1993); Kwon and Green (1994); Comai et al. (1994); Fukushima et al. (1998); Heller and Bengal (1998); Chang et al. (1999); Lee et al. (1999); Yuan and Gurley (2000); Čabart and Murphy (2001); Kamada et al. (2001); Hinkley et al. (2003); Friedman et al. (2007, 2008); Shah et al. (2009); Yang et al. (2014); Gouge et al. (2015)	Wood et al. (1998, 2000); Okamura-Oho et al. (1999); Shimohata et al. (2000); Yanagisawa et al. (2000); Nucifora et al. (2001); Feng et al. (2004); Zhang et al. (2006); Wang et al. (2008); Hou and Sibinga (2009); Ashkenazi et al. (2017)

SAGA, Spt-Ada-Gcn5- acetyltransferase. SNARE, soluble N-ethylmaleimide-sensitive factor activating protein receptor

**Fig. 1:**
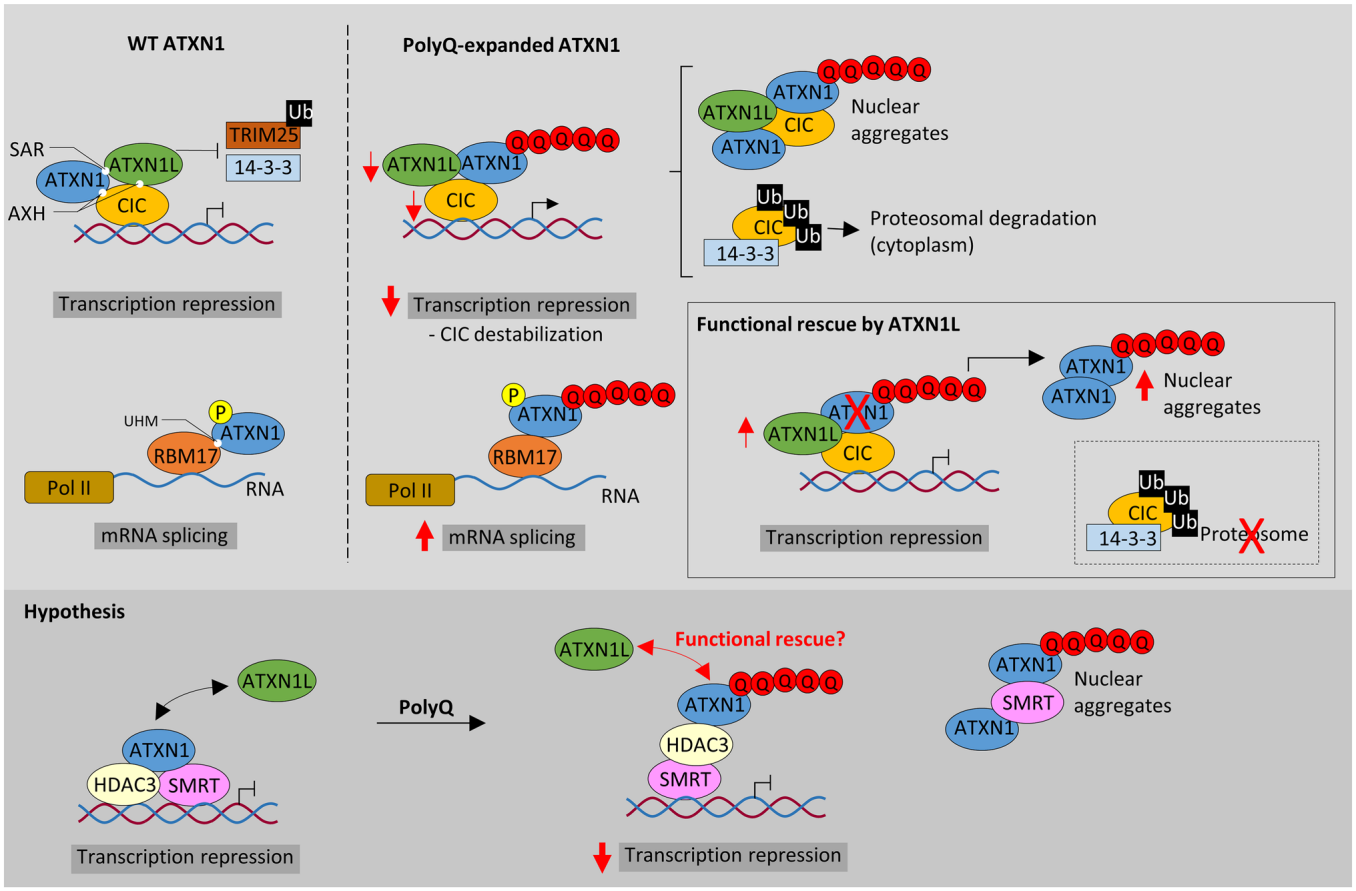
Model for SCA1 neuropathology and ATXN1/ATXN1L functional redundancy. ATXN1 has at least two distinct associated protein complexes in vivo: CIC-ATXN1 and ATXN1-RBM17. In the ATXN1 protein, it is believed that the AXH, SAR and UHM domains are interaction motifs for the transcriptional regulator CIC, ATXN1L and the RNA splicing factor RBM17, respectively. The CIC-ATXN1 complex details a synergistic relationship between the transcriptional repressor CIC, ATXN1, and ATXN1L to drive transcription repression. ATXN1–RBM17 modulates mRNA splicing through the phosphorylation state of ATXN1, but the polyQ-expanded ATXN1 favours the formation of RBM17-containing complexes contributing to SCA1 by means of a gain-of-function. At the same time, polyQ-expansions decreases the formation of ATXN1-CIC complexes, resulting in a partial loss-of-function. This event possibly results from CIC degradation by the E3-ligase TRIM25 and caspase 14-3-3 due to the lack of stabilization by ATXN1L in the complex. The mechanism causing the reduction of ATXN1L levels in SCA1 is still unknown. In SCA1 mice models, this functional overlap between ATXN1 and ATXN1L in the CIC-ATXN1 complex was demonstrated to partially rescue the polyQ-expanded ATXN1 function, contributing to pathology suppression. The increased ATXN1L levels competed with polyQ-expanded ATXN1 and normal ATXN1 (to a lesser extent) for association with CIC: decreasing the levels of polyQ-expanded ATXN1-containing CIC aggregates, promoting ATXN1 nuclear aggregation, and restoring the transcriptional function. In addition, ATXN1 and ATXN1L both interact with components of the co-repressor SMRT-HDAC3 complex but the polyQ-expanded ATXN1 seem to sequester SMRT components into nuclear inclusions (probably also affecting HDAC3 function as seen in MJD/SCA3). Thus, a functional rescue may be also possible in the context of the SMRT/HDAC3 transcription complex. UHM, U2AF homology domain. polyQ expansion (represented as Q in red). Pol II, RNA polymerase II (in dark yellow). Ub, ubiquitin (in black). P, phosphorylation (in yellow)

**Fig. 2:**
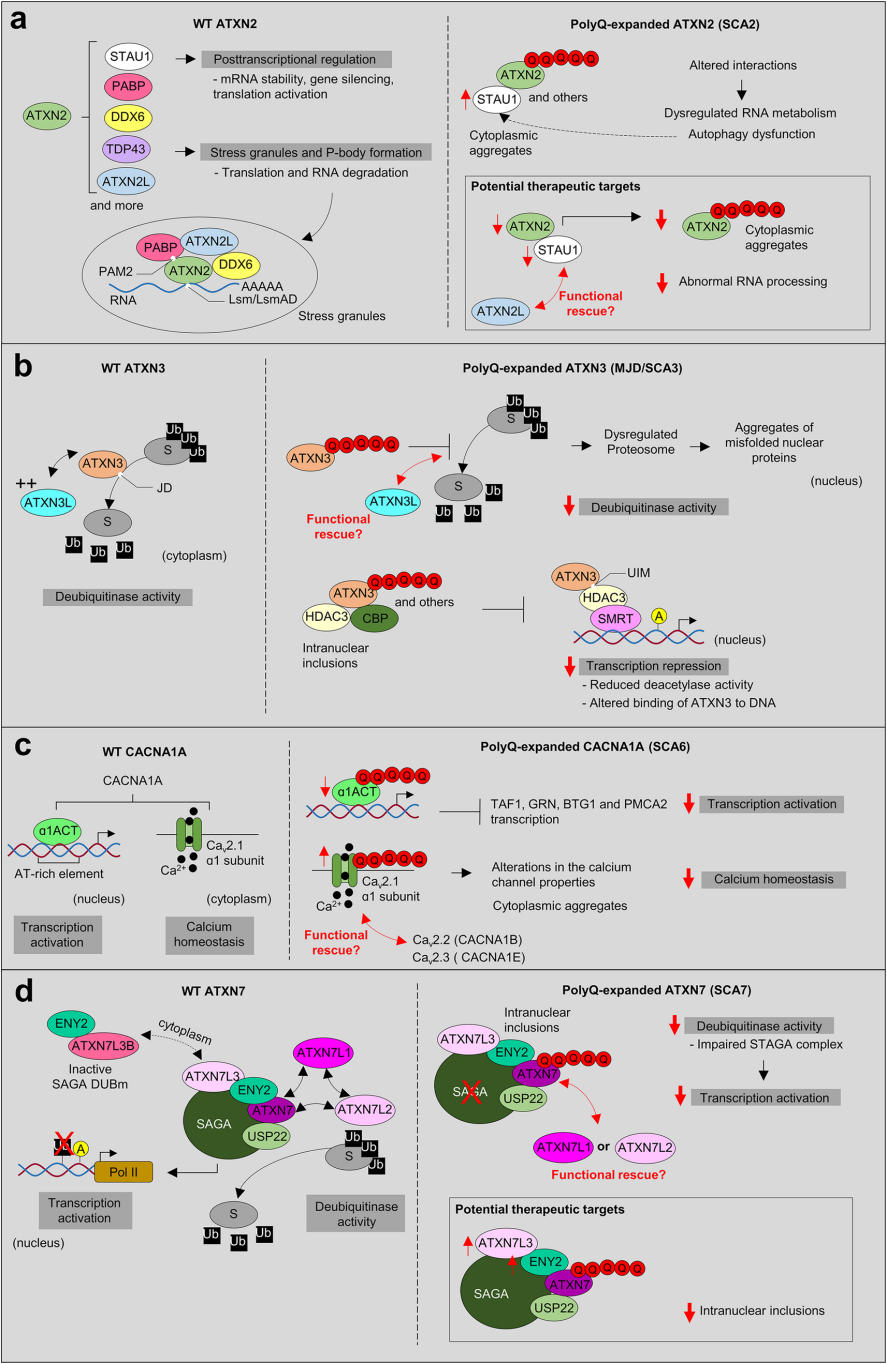

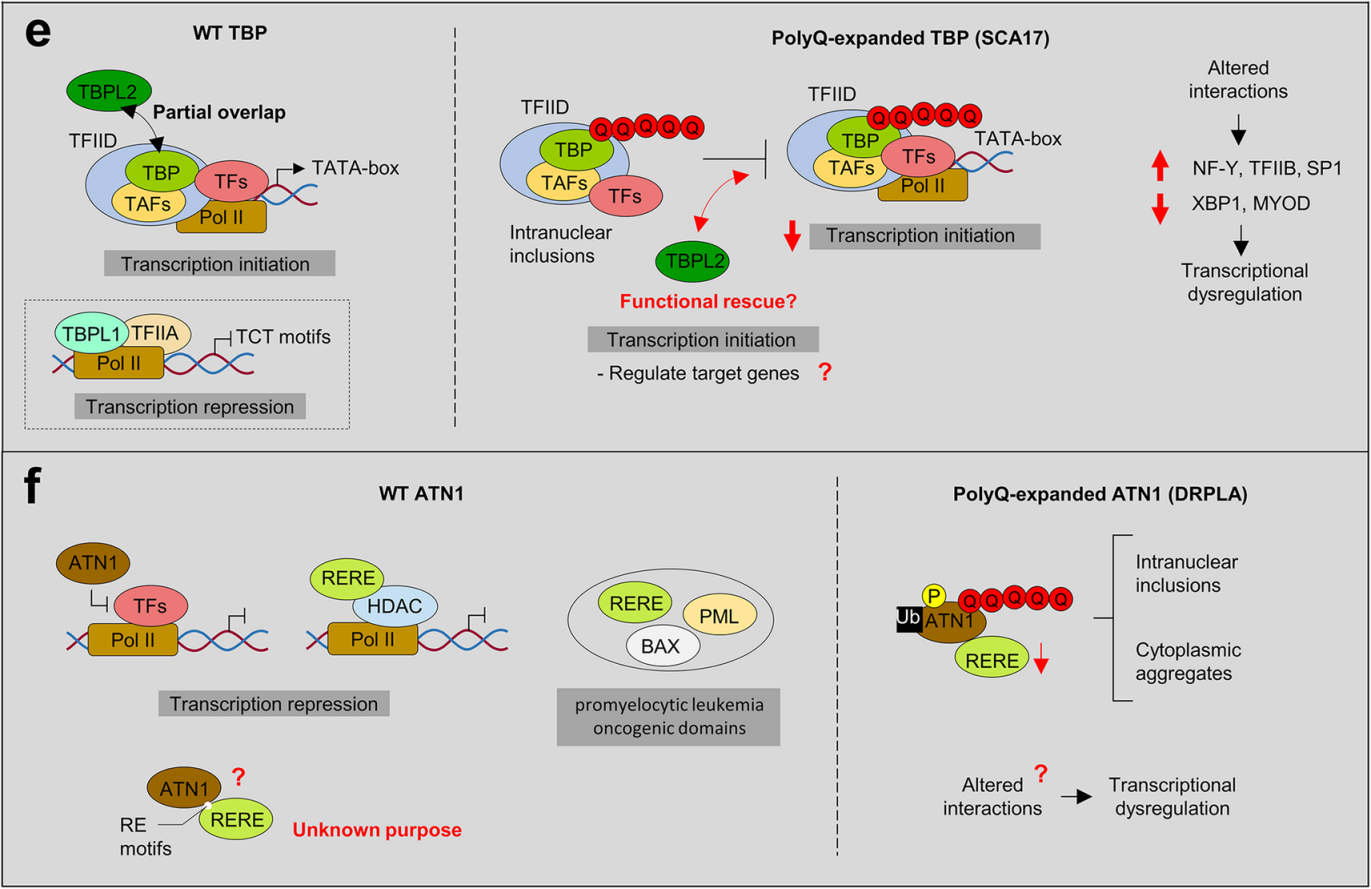
Molecular functions affected by expanded polyQ proteins in SCA2, SCA3, SCA6, SCA7, SCA17, and DRPLA. **a** ATXN2 interact directly or indirectly with numerous proteins implicated in RNA metabolism, as well as RNA itself, to regulate translation and stress granule/P-body formation. One hypothesis is that the polyQ-expanded ATXN2 inhibits its native interactions, impairing RNA metabolism and autophagy; thereby contributing to SCA2 pathogenesis. The exact mechanisms behind the alterations in RNA metabolism and stress granule/P-body formation in SCA2 remain unknown. Previous studies indicated that reduction of ATXN2 or STAU1, an interactor of ATXN2 and modulator of stress granules, was able to decrease aggregation of polyQ-expanded ATXN2 and improve motor behaviour in mice—suggesting that ATXN2L could also improve symptomatology in SCA2. **b** ATXN3 binds and cleaves polyubiquitin chains and has been implicated in ubiquitin-dependent protein quality control pathways (e.g., proteasome). Its paralog, ATXN3L Josephin domain revealed a higher deubiquitinase activity (+ +) possibly exhibiting functional redundancy. In MJD/SCA3, the polyQ-expanded ATXN3 seems to inhibit its deubiquitin activity and disrupt cellular proteostasis. Besides, it also can affect its DNA binding function and form nuclear inclusions with its transcription-related interactors (HDAC3, SMRT), impairing transcription repression. **c** CACNA1A gene encodes a bicistronic mRNA that produces two proteins: the membrane-localized α1A subunit of the Ca_v_2.1 channel and the transcription factor α1ACT. The polyQ-expanded CACNA1A leads to toxicity through impaired α1ACT-mediated transcription (decrease expression of gene targets—TAF1, GRN, BTG1 and PMCA2), as well as through altered Ca_v_2.1 channel properties. *CACNA1B* and *CACNA1E* genes also encode Ca_v_2 channels that could partially replace Ca_v_2.1 function in SCA6. **d** ATXN7 is a component of the SAGA transcription complex. PolyQ-expanded ATXN7 forms nuclear aggregates that are thought to sequester other components of the DUBm (ATXN7L3, ENY2, UPS22) such that the complex can no longer remove ubiquitin from its substrates. Even GCN5 histone acetyltransferase from the SAGA complex is sequestered into polyQ-expanded ATXN7 inclusions. Its paralog ATXN7L3B seems to also interact with ENY2 of the DUBm, however it seems to only function to limit SAGA activity by competing with ATXN7L3 (inactive SAGA). ATXN7L1 and ATXN7L2 have highly conserved domains with ATXN7 that appear to play interchangeable role in SAGA DUBm, however no studies characterized these proteins functions or interactions. Interestingly, co-overexpression of ATXN7L3 and ENY2 suppressed the formation of polyQ-expanded ATXN7 aggregates. **e** TBP is a transcription factor from the TFIID complex. The TBPL1 paralog does not bind to the TATA box as TBP, instead it relies on TCT motifs to repress transcription. In SCA17, the polyQ-expanded TBP impairs the DNA-binding and transactivation activity (targets—NF-γ, TFIIB, SP1, XBP1, MYOD) and exacerbates the formation of nuclear inclusions that reduce the TFIID complex availability for the transcription initiation process. As opposed to TBPL1, TBPL2 contains a highly conserved core domain and common interactors with TBP, constituting a promising candidate to compensate for TBP loss of function in SCA17. **f** ATN1 seems to work as a transcriptional co-repressor inhibiting transcription factors, but its function is not yet well understood. In DRPLA, the polyQ-expanded ATN1 was found to generate inclusions, in the cytoplasm and nucleus, which appear to disrupt ATN1 native protein–protein interactions. Also, polyQ-expanded ATN1 present abnormal ubiquitination, phosphorylation and cleavage in patients. The paralog RERE is thought to also be involved in transcription regulation and/or DNA binding, showing the ability to recruit histone deacetylases and serve as a transcriptional corepressor. In addition, RERE was proposed to be involved in caspase-mediated cell death (PML and BAX, promyelocytic leukemia oncogenic domains). Interestingly, RERE seems to directly interact with ATN1 partly via conserved RE motifs and form heterodimers, which can be related to the high tendency of this protein to aggregate with polyQ-expanded ATN1, likely depleting RERE protein in DRPLA patients. Still, not much information is known regarding these proteins nor DRPLA pathological mechanisms. TFs, transcription factors. TAFs, TBP-associated factors. HDAC, histone deacetylase. polyQ expansion (represented as Q in red). Pol II, RNA polymerase II (in dark yellow). Ub, ubiquitin (in black). A, acetylation (in yellow). P, phosphorylation (in yellow)

Unfortunately, the pathological mechanisms underlying SCAs remain poorly understood and only a few therapeutic approaches have been proposed to mitigate disease symptomatology (Klockgether et al. 2019). The abnormal polyQ expanded proteins increase the propensity for aggregation and misfolding processes, tending to reduce the interaction with usual binding partners and/or recruit other susceptible proteins into inactive cytoplasmic or nuclear inclusions. Consequently, these polyQ expansions cause a loss of function effect perturbing protein and RNA homeostasis in a specific manner dependent on the native protein function (Paulson et al. 2017; Lieberman et al. 2019) (Figs. 1 and 2). In contrast, these polyQ-expanded motifs can produce both expanded proteins and RNAs that promote abnormal interactions, *i.e.*, a gain of function mostly characterized by RNA toxicity, aberrant alternative splicing, repeat-associated non-AUG translation, and proteinopathy (Lieberman et al. 2019). In some cases, the native protein intermingles with the polyQ expanded form (*e.g.*, SCA1, MJD/SCA3) contributing to the aggregation process and loss of function effect, suggesting that a partial loss of function mechanism contributes to disease progression (Crespo-Barreto et al. 2010; Zeng et al. 2018). Moreover, in some SCAs, both the abnormal repeat expansions and single nucleotide variants appear to cause ataxia-like symptoms (*e.g.*, SCA6) with both gain of function and loss of function mechanisms underlying the observed phenotypes, emphasizing the complexity of SCAs etiology (Pietrobon 2002; Indelicato and Boesch 2021). The main challenge in developing treatments for SCAs is their genetic diversity and clinical variability. Studies have successfully reported the use of gene silencing strategies as therapies for some SCAs presenting a toxic gain of function mechanism (Bushart et al. 2016; McIntosh et al. 2021). Other strategies involve finding convergent mechanisms within multiple SCAs. Since many cerebellar ataxias seem to be caused by a process of protein aggregation, a promising treatment approach would implicate compounds that promote the redirection of protein aggregates to the proteasome, actively stimulating their degradation (Bushart et al. 2016; Klockgether et al. 2019).

### Paralog genes in humans

The current understanding of the SCA pathophysiological mechanisms may be improved through the analysis of paralogs, *i.e*., gene copies at different chromosomal locations that are derived from the duplication of a parental gene (Koonin 2005). Previous analyses of the human genome have predicted that at least 15% of human genes are duplicates (Li et al. 2001). These copies can arise from either DNA or RNA-based duplications. In the first case, parts of genes (*i.e.*, segmental duplication or small-scale duplication) can be copied via unequal crossing over and transposable elements, though the exact mechanisms are unknown. In the second case, RNA-based duplication (retrotransposition/retroduplication) can arise through the insertion of reverse transcribed mRNAs into the genome (Kaessmann 2010). It was first thought that retrotransposition events would consistently lead to inactive pseudogenes, *i.e.*, nonfunctional exonic sequences that cannot be expressed (Mighell et al. 2000). Still, retrogenes may become functional after the acquisition or evolution of regulatory elements to drive their expression (Casola and Betrán 2017).

Immediately after gene duplication, paralogs are expected to present complete functional redundancy, facilitating evolutionary change, but subsequently most degenerate and become pseudogenes by the accumulation of loss of function mutations (*i.e.*, pseudogenization/non-functionalization) (Innan and Kondrashov 2010). Exceptionally, paralogs accumulate mutations that can be fixed in a population and give rise to new advantageous functional properties (*i.e.*, neofunctionalization), or even partially conserve the ancestral function through gene-dosage amplification or subfunctionalization (duplication–degeneration–complementation model). The subfunctionalization process may reflect both neutral drift with complementary loss of function mutations between the paralogs making them indispensable for the ancestral function and adaptive evolution. Indeed, the ancestor function can become partitioned between the paralogs, and each may evolve toward the optimization of the retained function. The presence of duplicate genes with overlapping roles may relax/modify the selection pressure of the parental gene, while retaining a certain degree of functional overlap through long periods in evolution (Innan and Kondrashov 2010).

Consequently, paralogs may functionally compensate for the loss of function of parental genes in monogenic diseases and mask the phenotypic effects of their deleterious mutations (Kafri et al. 2006; Hsiao and Vitkup 2008). This would explain why disease-associated genes frequently have redundant paralogs which are conserved through generations (Dickerson and Robertson 2012; Chen et al. 2013). Therefore, investigating the relations between parental genes and their paralogs by an evolutionary approach would be crucial to expand our view on the functional relevance of these gene duplicates. Genomic evidence may elucidate about the rates of evolution and selective constraints among paralogs throughout reconstructed phylogenies. This way, it would be possible to identify the sites and/or regions of these genes that are under stronger selective constraint in humans. Nevertheless, as an initial exploration on this topic, frequency of genetic variants of the parental gene and its copies can be compared to hint their functional redundancy (gnomAD database; constraint metrics in Supplementary Table).

### Functional description of polyQ SCA-associated paralogs

#### SCA1: *ATXN1* gene

*ATXN1* (6p22.3) is a gene involved in transcriptional repression by interacting with several transcription regulators, *e.g.*, SMRT-HDAC3 repression complex, RBM17 splicing factor, and capicua (CIC) repressor complex (Tsai et al. 2004; Lam et al. 2006; Tong et al. 2011; Kim et al. 2014) (Fig. 1). The polyQ-expanded ATXN1 seems to promote the formation of abnormal protein interactions and nuclear toxic aggregates that perturb its capacity to regulate gene expression (Tejwani et al. 2021).

**Fig. 3:**
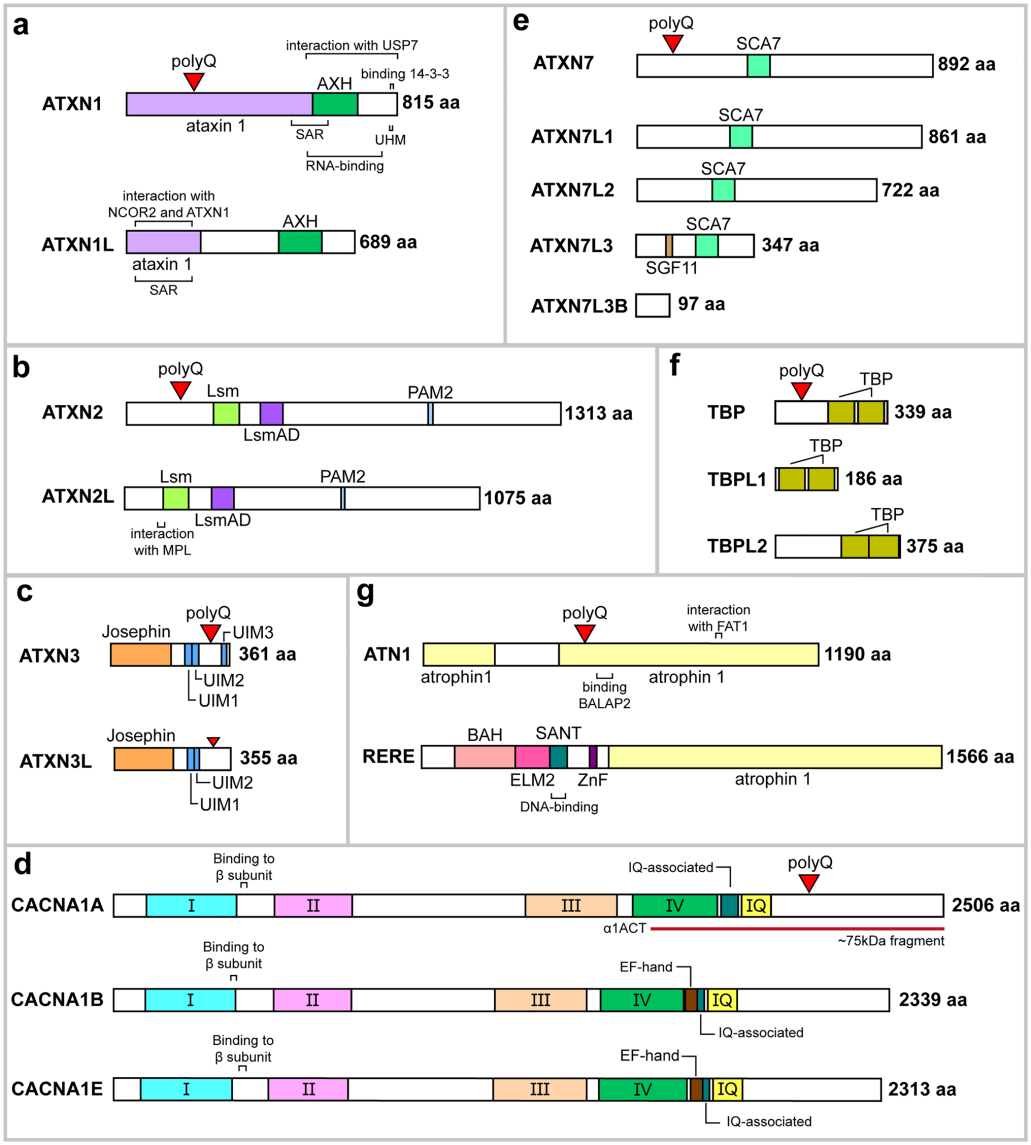
Domain architecture of polyQ SCA-associated proteins and their paralogs. In each case, a polyQ stretch is depicted at the approximate position (red triangle). **a** Both ATXN1 and ATXN1L contain the ataxin 1 and AXH domains, along with SAR regions. The AXH domain in both proteins is necessary for the interaction with CIC transcription repressor complex. Moreover, ATXN1 features a UHM motif to interact with the splicing factor RBM17 and a phosphorylation-dependent binding motif for the chaperone 14-3-3 near the C-terminus of the protein. **b** Both ATXN2 homologs contain Lsm and LsmAD domains for binding to RNA, and a PAM2 to interact with PABP and TDP43 transcription-related proteins. **c** ATXN3 long isoform contains an N-terminal catalytic Josephin domain and three UIM motifs, while ATXN3L has only two ubiquitin-binding sites like the shorter isoform of ATXN3. Interestingly, ATXN3L also contains a polyQ tract, but unlike ATXN3 it does not appear to expand, showing several interruptions (depicted in a small red triangle). **d** *CACNA1A* encodes for two proteins: Ca_v_2.1 voltage-gated calcium channel (full-length) and transcription factor α1ACT (C-terminal fragment) by using a bicistronic mRNA with an internal ribosomal entry site. CACNA1A along with CACNA1B and CACNA1E contain four conserved homologous domains (I–IV), each with six transmembrane segments, and IQ/IQ-associated to bind to CaM. The two paralogs additionally present EF-hand domains responsible for calcium binding. **e** ATXN7 and its paralogs contain a ZnF domain SCA7 essential for ATXN7 association to DUBm from STAGA (excluding ATXN3LB). ATXN7L3 shows another ZnF/SGF11 domain that plays an important role in the DUB activity and DNA-binding. **f** All TBP homologs consist of two TBP domains to bind to a DNA sequence called TATA-box. **g** ATN1 protein only contains two atrophin 1 domains while its paralog RERE (long isoform; -L) includes four specific domains in the N-terminal (BAH, ELM2, SANT, and ZnF/GATA) besides atrophin 1 domain, possibly conferring specific properties. RERE-S isoform simply consist of the atrophin 1 N-terminal domain. UHM, U2AF homology domain. UIM, ubiquitin interacting motifs. IQ, IQ-like CaM interaction domain. CaM, Calmodulin. Amino acid numbering is based on Uniprot accession numbers P54253 (ATXN1), P0C7T5 (ATXN1L), Q99700 (ATXN2), Q8WWM7 (ATXN2L), P54252 (ATXN3), Q9H3M9 (ATXN3L), O00555 (CACNA1A), Q00975 (CACNA1B), Q15878 (CACNA1E), O15265 (ATXN7), Q9ULK2 (ATXN7L1), Q5T6C5 (ATXN7L2), Q14CW9 (ATXN7L3), Q96GX2 (ATXN7L3B), P20226 (TBP), P62380 (TBPL1), Q6SJ96 (TBPL2), P54259 (ATN1), RERE-L(Q9P2R6), and RERE-S (Q9P2R6-2)

Previous research refers to the existence of one *ATXN1* paralog frequently called ataxin 1 like (*ATXN1L*) or brother of ataxin 1 (*BOAT*; 16q22.2) (Mizutani et al. 2005; Bowman et al. 2007), which likely originated from a DNA-based duplication mechanism (Table 2). *ATXN1L* transcripts were widely detected in human cell lines and tissues, with the highest expression levels found in the cerebellum and the cerebral cortex (Mizutani et al. 2005). Interestingly, *ATXN1L* was proposed as a candidate gene in an ataxic patient with early cerebellar dysfunction (Monies et al. 2017), as well as a regulator of hematopoietic stem cells quiescence/proliferation (Kahle et al. 2013). Still, ATXN1L function remains poorly understood. At the protein level, ATXN1 and ATXN1L are highly conserved, especially at the ATXN1 and HMG-box protein 1 interacting (AXH) domains (66% homology), showing a global homology of 23–33% (Mizutani et al. 2005; Carlson et al. 2009; Vauti et al. 2021) (Fig. 3a). Thus, based on their structural similarity and tissue expression patterns, it was hypothesized that ATXN1 and ATXN1L are likely to participate in related biological pathways (Bowman et al. 2007). These homologous proteins were shown to interact with each other and share some binding partners, including the transcriptional repressor CIC (Lam et al. 2006), SMRT-HDAC3 complex (Tsai et al. 2004; Mizutani et al. 2005), and a SMRT-associating transcription factor from the Notch pathway (CBF1) (Tong et al. 2011). Indeed, ATXN1L was demonstrated to inhibit *NOL3* and *PYDC1* expression via HDAC3 and CIC complexes, respectively, mediating apoptosis and pyroptosis in cardiomyocytes (Cai et al. 2022; Xu et al. 2022). Moreover, ATXN1L-CIC transcriptional repressor was found to regulate the expression of ETS-domain transcription factors, which modulated drug resistance in *RAS*-mutant cancers (Wang et al. 2017).

**Table 2:** Description of human paralogs of genes involved in polyQ SCAs reviewed in this study

Disease	Paralog	Chromosome location	Origin	RNA expression	Phenotype	Function	References
SCA1	*ATXN1L*	16q22.2	DNA-based duplication	Mainly expressed in brain (higher in gonads and lymphoid tissue)**	Development (brain, lung)	Transcription regulator (CIC-ATXN1 complex)	Tsai et al. (2004); Mizutani et al. (2005); Lam et al. (2006); Carlson et al. (2009); Tong et al. (2011); Vauti et al. (2021)
SCA2	*ATXN2L*	16p11.2	DNA-based duplication	Widely expressed at higher levels in testis	Unknown	Stress grabule assembly (mRNA metabolism)	Meunier et al. (2002); Figueroa and Pulst (2003); Kaehler et al. (2012, 2015); Lee et al. (2018)
MJD/ SCA3	*ATXN3L*	Xp22.2	Retrotransposition	Only detected in testis	Unknown (related to cell proliferation/ migration)	Protein deubiquitinase	Rodrigues et al. (2007); Schmitt et al. (2007); Buus et al. (2009); Weeks et al. (2011); Ge et al. (2015); Sousa e Silva et al. (2023)
	*ATXN3L2*	8q23.2	Retrotransposition (interrupted ORF)	Unknown	Unknown	Unknown (possibly a processed pseudogene/mRNA)	–
SCA6	*CACNA1B*	9q34	DNA-based duplication	Mainly detected in brain, pituitary gland, and testis	Development (brain)	High voltage-gated calcium channel	Westenbroek et al. (1992; Wheeler et al. (1994); Catterall et al. (2005); Nakagawasai et al. (2010); Heyes et al. (2015); Gorman et al. (2019)
	*CACNA1E*	1q25.3	DNA-based duplication	Mainly detected in brain	Unknown (related to synaptic plasticity)	High Voltage-gated calcium channel	Day et al. (1996); Ophoff et al. (1996); Dietrich et al. (2003); Catterall et al. (2005); Parajuli et al. (2012); Heyes et al. (2015)
SCA7	*ATXN7L1*	7q22.3	DNA-based duplication	Widely expressed at higher levels in testis	Unknown	Protein binding (SAGA DUBm)	Vermeulen et al. (2010); Helmlinger and Tora (2017)
	*ATXN7L2*	1p13.3	DNA-based duplication	Widely expressed at higher levels in skeletal muscle (brain, pituitary gland, and testis)*	Unknown	Unknown (possibly similar to ATXN7L1)	Vermeulen et al. (2010); Helmlinger and Tora (2017)
	*ATXN7L3*	17q21.31	DNA-based duplication	Widely expressed at higher levels in brain	Unknown	Transcription regulator/protein binding (SAGA complex)	Helmlinger et al. (2004); Bonnet et al. (2010); Lang et al. (2011)
	*ATXN7L3B*	12q21.1	Retrotransposition	Widely expressed at higher levels in brain and lymphoid tissue (female gonads)*	Unknown	Transcription regulator (transcription factor, lncRNA)	Tan et al. (2014); Li et al. (2016b)
SCA17	*TBPL1*	6q23.2	DNA-based duplication	Widely expressed at higher levels in testis	Spermatogenesis	Transcription initiation factor (TATA-less genes)	Moore et al. (1999); Ohbayashi et al. (1999b, a); Rabenstein et al. (1999); Martianov et al. (2001); Zhang et al. (2001); Isogai et al. (2007); Duttke et al. (2014); Kedmi et al. (2014); Wang et al. (2014)
	*TBPL2*	14q22.3	DNA-based duplication	Mainly detected in brain and skeletal muscle (testis)**	Oogenesis, muscle differentiation	Transcription initiation factor (TATA-box promoters)	Müller et al. (2001); Persengiev et al. (2003); Bártfai et al. (2004); Jallow et al. (2004); Gazdag et al. (2007, 2009); Akhtar and Veenstra (2009); Malecova et al. (2016)
DRPLA	*RERE*	1p36.23	DNA-based duplication	Widely expressed at higher levels in brain and muscle tissue (female gonads)*	Development (cerebellum)	Transcription factor (histone deacetylases recruitment)	Onodera et al. (1995); Yanagisawa et al. (2000); Waerner et al. (2001); Asai et al. (2006); Wang et al. (2006, 2008); Plaster et al. (2007); Shen et al. (2007); Shen and Peterson (2009)

ORF, open reading frame

In case gene expression data reported in the literature is only partially supported* or discordant** with databases, the respective additional or alternative tissues are in parentheses. GTEx (http://gtexportal.org/home/); The Human Protein Atlas (http://www.proteinatlas.org/); FANTOM (http://fantom.gsc.riken.jp/)

Among all the identified interactors, the synergistic relationship described between ATXN1, ATXN1L, and CIC (CIC-ATXN1 complex) is of particular interest because these are the only interactors whose protein levels are significantly reduced in SCA1 models (Mizutani et al. 2005; Lam et al. 2006). In fact, both *ATXN1* and *ATXN1L* knock-out (KO) resulted in early perinatal lethality and several developmental abnormalities in mice, but when comparing to the double KO model, ATXN1 seemed to partially compensate for the loss of ATXN1L function (Lee et al. 2011). ATXN1 polyQ-expansion was demonstrated to alter protein conformation, leading to abnormal interactions (Lim et al. 2006, 2008; Rocha et al. 2019) and functional defects in the CIC-ATXN1 complex, probably due to an apparent reciprocal functional relationship (Lam et al. 2006; Fryer et al. 2011; Wong et al. 2018) (Fig. 1). ATXN1L levels were positively associated with both the CIC expression (Crespo-Barreto et al. 2010; Wang et al. 2017; Wong et al. 2018) and the aggregation of polyQ-expanded ATXN1 (Lam et al. 2006), indicating an apparent relation between the complex formation and SCA1 pathology. Interestingly, ATXN1-CIC interaction was shown to be critical for SCA1 pathogenesis in Purkinje cells, while its ablation partially improved the neurological phenotype in mice (gain of function mechanism) (Rousseaux et al. 2018; Coffin et al. 2023). Moreover, in SCA1 the polyQ-expanded ATXN1 (Ser776 phosphorylation by PKA) favours the formation of RBM17-containing complexes (related to mRNA splicing), inspiring a gain of function mechanism (Kim et al. 2014) (Fig. 1). In fact, in vivo inhibition of ATXN1 Ser776 phosphorylation enhanced degradation of ATXN1 and delayed the onset of ataxia in SCA1 mice (Pérez Ortiz et al. 2018). Several studies in Drosophila and mouse models support the hypothesis of interchangeability between ATXN1L and ATXN1 in the CIC-ATXN1 complex and SCA1 pathology. These findings mostly support that ATXN1L is able to modulate the cytotoxicity of polyQ-expanded ATXN1 and suppress SCA1 symptomatology by reducing the spontaneous formation of expanded ATXN1-CIC complexes and/or compensating for ATXN1 native function by competing for the interaction with CIC (Mizutani et al. 2005; Bowman et al. 2007; Crespo-Barreto et al. 2010; Carrell et al. 2022). Indeed, CIC is polyubiquitinated and degraded by the E3 ubiquitin ligase TRIM25 in the absence of ATXN1L stabilization, manifesting the dominant role of ATXN1L (Wong et al. 2020). Also, the targeting of ATXN1 (self-association region, SAR; Fig. 3a) by ATXN1L could be suppressing SCA1 cytotoxicity (Mizutani et al. 2005) (Fig. 1). Simultaneously, this implies that SCA1 is induced by the formation of toxic hypermorphic complexes that subsequently affect the transcriptional repression activity of the ATXN1-CIC complex, including ATXN1L (Lasagna-Reeves et al. 2015; Lu et al. 2017; Rousseaux et al. 2018).

#### SCA2: *ATXN2* gene

*ATXN2* (12q24.12) encodes for a protein involved in mRNA degradation, stability, and translation by modulating the PABP protein, 3′ untranslated regions (Lee et al. 2018), and stress granule and P-body formation (Nonhoff et al. 2007). The polyQ-expanded ATXN2 causes toxic accumulation of cytoplasmic protein aggregates, inhibiting its native interactions and causing transcription/translation dysregulation (Egorova and Bezprozvanny 2019) (Fig. 2a).

The paralog ataxin 2 like (*ATXN2L* or *A2RP*; 16p11.2), possibly derived from an *ATXN2* DNA-based duplication event (Table 2), encodes a protein with 50% homology. These paralog proteins present highly similar functional domains [like-sm (Lsm), like-Sm-associated domain (LsmAD), and PABP interacting motif (PAM2); Fig. 3b], and two known common interactors with ATXN2: PABP and DDX6 (Figueroa and Pulst 2003; Jiménez-López and Guzmán 2014) (Fig. 2a). In terms of expression, this paralog was found to be mainly detected in testis (Meunier et al. 2002), with a weak but widespread expression in human adult brain (mostly in the cerebellum) (Figueroa and Pulst 2003). Both ATXN2 and ATXN2L present a proline-rich domain, a conserved region responsible for mediating the direct interaction with SH3 motifs, located in components of the growth factor receptor endocytosis apparatus (Nonis et al. 2008; Lin et al. 2019). ATXN2L was found to be in a complex along PABP, DDX6 and ATXN2 in stress granules and associated with nuclear splicing machinery, suggesting functional redundancy (Kaehler et al. 2012) (Fig. 2a). Besides, ATXN2L was shown to play a major role in the in vitro formation of cytoplasmic granules and P-bodies in mammalian cells, suggesting that it could be important for the nucleation step of stress granules (Kaehler et al. 2012). This functional difference could result from differential methylation applied to *ATXN2* and *ATXN2L* by PRMT1 and/or differences in the C-terminal region, since ATXN2L comprises a sequence absent in ATXN2 that shows homology to a protein family involved in P-body formation (Kaehler et al. 2012, 2015). Still, the implications of ATXN2L promoting the formation of stress granules remain unclear. Recently, a protein–protein interaction network analysis of genes affected by rare and/or potentially pathogenic variants in a member of a large SCA1 family suggested the interaction of ATXN1 with ATXN3, ATXN7, and ATXN2L. This study proposed that genetic variation in *ATXN2L* may play an additive role and exacerbate the neuropathology already driven by the abnormal intermediate polyQ-expanded ATXN1 (Morello et al. 2020). Studies demonstrated that an *ATXN2* KO mouse model is associated with a metabolic syndrome, involving hypercholesterolemia and diabetes mellitus, possibly related with a post-transcriptional effect of ATXN2 on the insulin receptor expression in liver and in cerebellum (Lastres-Becker et al. 2008). On the other hand, ATXN2L absence triggered mid-gestational embryonic lethality in homozygous KO mice, but no consequent dysregulation was seen for ATXN2 (Key et al. 2020). These findings reinforce the crucial role of ATXN2L for RNA metabolism and translation, leaving in open ATXN2L and ATXN2 parallel contribution to SCA2.

A recent study indicated that reduction of ATXN2 or STAU1, a interactor of ATXN2 and modulator of stress granules, was able to decrease aggregation of polyQ-expanded ATXN2 and improve motor behaviour in transgenic mice (Paul et al. 2018) (Fig. 2a). Thus, ATXN2L could also have a similar effect since it is known to regulate stress granules. Further research will help  to unravel if ATXN2L can eventually remediate ATXN2 native functions and/or interactions, as well as be involved in SCA2 pathogenesis.

#### MJD/SCA3: *ATXN3* gene

*ATXN3* (14q32.12) has been associated with transcription regulation by binding to DNA and interacting with several transcription-related factors (*e.g.*, CBP, P300 histone acetyltransferase, HDAC3, and SMRT) (Li et al. 2002; Evert et al. 2006), and also with proteostasis via ubiquitin–proteasome system (Burnett et al. 2003; Burnett and Pittman 2005). The polyQ-expanded ATXN3 causes conformational changes that promote intraneuronal nuclear inclusions and cytotoxicity, which inhibit its function (deubiquitinase and DNA binding) and native protein interactions (Da Silva et al. 2019). Also, these nuclear aggregates hinder transcription related proteins such as HDAC3 and CBP, impairing transcription repression (McCampbell et al. 2000; Evert et al. 2006) (Fig. 2b).

*ATXN3* has two paralogs identified: ataxin 3 like (*ATXN3L;* Xp22.2) that probably resulted from a recent retrotransposition event in primates (Scheel et al. 2003; Vlasschaert et al. 2017; Sousa e Silva et al. 2023) and *LOC100132280* (herein called *ataxin 3 like 2*, *ATXN3L2*; 8q23.2), which reveals a premature stop codon that likely produces an inactive processed pseudogene (Table 2). The fact that *ATXN3L2* is most likely not translated into protein does not rule out the hypothesis of *ATXN3L2* mRNA playing a role in gene expression regulation. Nevertheless, as little is known about the possible role of *ATXN3L2* and the presence of its transcript in the brain, we will further focus on the known function and interactions of *ATXN3L* with its parental gene.

ATXN3L and ATXN3 showed similar Josephin domains with a high degree of sequence identity (85%) (Weeks et al. 2011), suggesting that both proteins might play similar functions (Fig. 3c). *ATXN3L* also contains a (CAG)_n_ tract but unlike *ATXN3* it does not appear to expand, showing several GAA interruptions encoding for glutamic acid (Weeks et al. 2011). *ATXN3* and *ATXN3L* have shown different expression patterns (with the retrogene mainly present in testis) and distinct ubiquitin recognition modes, which may be physiologically relevant (Weeks et al. 2011; Sousa e Silva et al. 2023). Nevertheless, in vitro experiments demonstrated that both ATXN3 and ATXN3L can cleave Lys-48-linked and Lys-63-linked polyubiquitin chains, with the Josephin domain of ATXN3L revealing higher deubiquitinase activity and functional redundancy (Fig. 2b). Intriguingly, three mutations (S12F, R59L, and T60A; called triple mutation) occurring in ATXN3 were found to almost equalize the corresponding deubiquitinase activity of ATXN3L, proposing that ATXN3 was subjected to evolutionary constraints (Weeks et al. 2011). In addition, *ATXN3* KO animal models (mouse and *C. elegans*) had no overt phenotype mainly showing alterations in the ubiquitin–proteasome pathway (Rodrigues et al. 2007; Schmitt et al. 2007), which could probably be reverted by ATXN3L.

So far, the role of ATXN3L remains poorly characterized, but some studies have suggested a potential role in cell proliferation and migration possibly by preventing the degradation of proteins like KLF5 (Buus et al. 2009; Ge et al. 2015), also being possibly involved in premature ovarian failure by promoting oocyte maturation (Lee et al. 2020). Interestingly, *ATXN3L* was linked to sporadic Alzheimer’s disease, but its relation remain unclear (Gómez-Ramos et al. 2015). In addition, ATXN3L seems to regulate directly or indirectly *PTEN* transcription, similar to ATXN3 and Josephin domain containing 1 (JOSD1) proteins, possibly indicating a conserved function in the Josephin domain-containing proteins (Sacco et al. 2014).

#### SCA6: *CACNA1A* gene

*CACNA1A* (19p13.13) is involved in neuronal signal transmission in neurons presynaptic terminals, encoding for the pore-forming α1A subunit of P/Q type calcium voltage-gated channels (Ca_v_) 2.1, mainly expressed in the cerebellum (specially in Purkinje cells) (Catterall 2000; Catterall et al. 2005). The polyQ-expanded CACNA1A increases the formation of mainly cytoplasmic protein inclusions, reduces Ca^2+^ influx into Purkinje cells in vitro, and impairs transcriptional dysregulation possibly leading to neurodegeneration (Giunti et al. 2015) (Fig. 2c). *CACNA1A* is also known to induce episodic ataxia type 2, suggesting that even missense variants in crucial protein domains can cause ataxia-related symptoms (Indelicato and Boesch 2021). In fact, KO mice display severe ataxia and develop absence seizures (Jun et al. 1999; Fletcher et al. 2001) similarly to some mouse strains with missense mutations, which suffer from ataxia and seizures, showing reduced P/Q type currents in Purkinje cells (Pietrobon 2002). Moreover, conditional KO mouse with a postnatal deletion of *CACNA1A* in Purkinje cells do not initially present ataxia and absence epilepsy but instead develop them during adulthood (Mark et al. 2011), which propose these disorders arise from defects beginning in late infancy—an opportunity window for therapy.

Interestingly, *CACNA1A* is a bicistronic gene, which means a single transcript can give rise to two independent proteins: (a) the α1A subunit of the Ca_v_2.1 channel has a short and a long splice transcripts but only the latter contains the CAG repeat, and (b) the α1ACT transcription factor which shares the C-terminal sequence of the α1A subunit due to an internal ribosome entry site element (Du et al. 2013) (Figs. 2c and 3d). Thus, two different proteins contain a polyQ tract, the long splice transcript of the α1A channel subunit and the transcription factor α1ACT, both selectively expressed in cerebellar Purkinje cells (Bavassano et al. 2017; Du et al. 2019; Govek and Hatten 2019). In particular, the polyQ-expanded α1ACT leads to ataxia and cerebellar atrophy in SCA transgenic mice, being essential for neonatal neuronal development and neurite outgrowth, since it lacks transcription factor function (TAF1, GRN, BTG1 and PMCA2 target genes; Fig. 2c) and causes cell death (Du et al. 2013, 2019). Moreover, α1ACT transcription factor was also impaired in α1A/Ca_v_2.1 homozygous KO mice, though the expression of normal α1ACT in Purkinje cells leads to a slight phenotypic improvement, they still remain non-viable past the neonatal period (Du et al. 2013). Interestingly, decreased expression of α1ACT but increased expression of Ca_v_2.1 was observed in patient-derived Purkinje cells (Ishida et al. 2016) (Fig. 2c).

Although 26 *CACNA1A* paralogs are annotated in the human genome, only *CACNA1B-I* and *CACNA1S* genes also encode pore-forming α1 subunits of Ca2 + voltage-gated channels (Ca_v_1, Ca_v_2, or Ca_v_3) (Catterall et al. 2005; Heyes et al. 2015). Sodium voltage-gated channel alpha subunit 1–11 (*SCN1A-11A*) genes encode for α subunits of sodium voltage-gated channels (Na_v_1.1–1.9), excepting *SCN7A* (Na_x_), which has lost its voltage-gated function and rapidly evolved as a signal transducer of extracellular Na^+^ ions (De Lera and Kraus 2015; Dolivo et al. 2021). Sodium leak channel, non-selective (*NALCN*) gene encodes a channel that regulates the resting membrane potential and neuronal excitability as part of a complex including G protein-coupled receptors (Cochet-Bissuel et al. 2014). Two pore segment channel 1 and 2 (*TPCN1* and *TPCN2*) genes encode for channels responsible for the acid adenine dinucleotide phosphate (NAADP)‐mediated calcium release within the endo-lysosomal system (Pitt et al. 2016). Finally, cation channel sperm associated 1–4 (*CATSPER1-4*) genes were first identified based on sequence similarity to the Ca_v_1.3 channel and encode for channels with a crucial role in sperm physiology and fertility (Shukla et al. 2012). Even though *SCNA*, *NALCN*, *TPCN* and *CATSPER* groups of paralogs have distinct functions and properties related to ion channels, only the closely related *CACNA1* gene family (with 10 homologs, duplicated in vertebrates) can show similar physiological functions (Lagman et al. 2013; Abascal et al. 2015). In particular, genes classified inside each family of voltage-gated Ca^2+^ channels are evolutionarily closer (Pietrobon 2002; Catterall et al. 2005), making *CACNA1B* and *CACNA1E* good candidates to explore functional redundancy since they also belong to the Ca_v_2 subfamily (Figs. 2c and 3d; Table 2).

*CACNA1B* (9q34.3) encodes for another pore-forming α1B subunit of the pre-synaptic neuronal Ca_v_2.2 channel of N-type, responsible for the generation of high voltage-activated Ca^2+^ current with moderate voltage-dependent inactivation (Catterall et al. 2005). CACNA1B has been found to mostly participate in neuronal processes related to neuronal growth, synaptic function, and development (Heyes et al. 2015). In fact, CACNA1B is widely distributed throughout the central and peripheral nervous system (Westenbroek et al. 1992; Wheeler et al. 1994) while CACNA1A was reported to be particularly prevalent in the cerebellum (Ophoff et al. 1996). Previous studies reported a functional overlap between CACNA1A and CACNA1B, both being responsible for most of the glutamatergic neurotransmission in hippocampal synapses (Cao et al. 2004). Interestingly, CACNA1B coordination with CACNA1A seems to be crucial to control synaptic transmission throughout the nervous system. CACNA1B is thought to be crucial for neurotransmission in the early postnatal period as it is replaced by CACNA1A in mature synapses during neurodevelopment (Scholz and Miller 1995; Gorman et al. 2019), highlighting that the contribution of the two homologs is time specific (Iwasaki et al. 2000). In addition, both *CACNA1A* and *CACNA1B* mRNAs are regulated by the Nova-2 splicing factor, which preferentially selects certain isoforms (e24a over e31a splicing isoform) to be expressed in the brain (Allen et al. 2010). A study demonstrated that homozygous *CACNA1B* KO mice only showed mild phenotype (sympathetic nerve dysfunction) when a lethal effect was expected (Ino et al. 2001). However, later *CACNA1B* was shown to be essential for correct neurodevelopment and predicted to be highly intolerant to loss of function variants, as demonstrated by debilitating symptoms such as memory impairment and atypical locomotor activity in homozygous KO mice (Nakagawasai et al. 2010; Gorman et al. 2019). Besides, single nucleotide and copy-number variants involving *CACNA1B* have been described in individuals with neurovascular disorders and schizophrenia, and implicated in developmental and epileptic encephalopathies (Gorman et al. 2019). For instance, a *CACNA1B* missense variant (c.4166G > A; p.Arg1389His) was reported in a family with adult-onset myoclonus-dystonia and cardiac arrhythmia, which resulted in lower current Ca_v_2.2 channels, emphasizing the functional relevance of this paralog (Groen et al. 2015).

*CACNA1E* (1q25.3) encodes for the moderate R-type α1E subunit of the Ca_v_2.3 channel, generating Ca^2+^ current with fast voltage-dependent inactivation (Catterall et al. 2005), known for its role in synaptic plasticity (Dietrich et al. 2003; Heyes et al. 2015). Previous studies refer that *CACNA1E* is especially distributed throughout the central nervous system as *CACNA1A* (Day et al. 1996; Ophoff et al. 1996; Parajuli et al. 2012). *CACNA1E* variants have been linked to several neurological disorders, including developmental and epileptic encephalopathy, autism spectrum disorder, and migraine (Helbig et al. 2018; Heyne et al. 2018; Takata et al. 2019; Royer-Bertrand et al. 2021), but has not been established as a disease-causing gene. In contrast with *CACNA1B*, *CACNA1E* homozygous KO mice did not reveal any evident neurological symptoms, but showed abnormalities in pain responses due to an attenuation of the inhibitory effect in the descending anti-nociceptive pathway (Saegusa et al. 2000).

#### SCA7: *ATXN7* gene

*ATXN7* (3p14.1) encodes for a transcriptional factor comprised in the multiprotein Spt-Ada-Gcn5-acetyltransferase (SAGA) complex, which regulates transcription through its histone-modifying enzymes, histone acetyltransferase GCN5, and ubiquitin-specific protease USP22 (Cornelio-Parra et al. 2021). The ATXN7 protein serves to anchor the SAGA’s deubiquitinase module (DUBm) to the larger core module (Lee et al. 2009; Ellisdon et al. 2010), participate in the regulation of histone acetylation/deubiquitination (Lang et al. 2011), and facilitate the RNA polymerase II recruitment (Bonnet et al. 2014). Moreover, ATXN7 may be involved in microtubule stabilization (Nakamura et al. 2012). The polyQ-expanded ATXN7 seems to recruit other factors like the SAGA’s DUBm proteins [ENY2 transcription and mRNA export factor, USP22, and the paralog ataxin 7 like 3 (ATXN7L3)] into nuclear aggregates. Consequently, these aggregates prevent the adequate formation of DUBm, altering the deubiquitinase activity of SAGA and chromatin state of a subset of genes probably related to retina function (one of the main affected regions in SCA7) (Mohan et al. 2014; Lan et al. 2015; Goswami et al. 2022). Yet, the co-overexpression of ATXN7L3 and ENY2 enabled to mitigate the effect of ATXN7 polyQ inclusions (Lan et al. 2015) (Fig. 2d). Interestingly, HDAC3 was found to physically interact with ATXN7, leading to its increased stability, subcellular localisation and post-translational modifications (Duncan et al. 2013). Besides, HDAC3 was highly expressed in both neurons and glia in the cerebellum of non-transgenic and SCA7 transgenic mice, suggesting a role in SCA7 neuropathology (Duncan et al. 2013).

So far, four paralogs of *ATXN7* have been identified: ataxin 7 like 1, 2, 3 and 3B [*ATXN7L1* (7q22.3), *ATXN7L2* (1p13.3), *ATXN7L3* (17q21.31), and *ATXN7L3B* (12q21.1)] (Helmlinger et al. 2004) (Table 2). ATXN7, ATXN7L1, and ATXN7L2 proteins share three conserved regions, designated as domains I (C2H2 zinc-finger), II (SCA7), and III (C-terminal), revealing a high degree of homology (≥ 50%) that suggests functional redundancy (Fig. 3e). In contrast, ATXN7L3 only shares the first two domains with ATXN7 (≤ 35% homology) and presents a distinct SCA7 domain that probably diverged over evolution to achieve specific functions in the SAGA complex, since it was suggested to be originated from an ancient duplication of *ATXN7* (Helmlinger et al. 2004; Bonnet et al. 2010). In concordance, ATXN7L3 was shown to play a central and non-redundant function in the SAGA complex, contrarily to the remaining homologs (Zhao et al. 2008; Lang et al. 2011) (Fig. 2d), weakening the hypothesis of functional overlap between ATXN7 and ATXN7L3. Simultaneously, no alternative human proteins substituting ATXN7L3 function were ever described, which suggests that the loss of ATXN7L3 function would exclusively impair SAGA function (Zhao et al. 2008). *ATXN7L3B* resulted from the retrotransposition of the *ATXN7L3* gene (Table 2), both sharing 74% of identity in their correspondent N-terminal regions (including the ENY2-binding region), thereby suggesting common interactors shared by the two homologs (Li et al. 2016b). However, ATXN7L3B localizes in the cytoplasm and seems to solely interact with ENY2 in the SAGA complex, and unlike the ATXN7L3 DUBm, the ATXN7L3B complex cannot function efficiently in vitro. Therefore, the ATXN7L3B-ENY2 interaction could be limiting SAGA activity by sequestering ENY2 in the cytoplasm and competing with ATXN7L3 (Fig. 2d). Moreover, ATXN7L3 and ATXN7L3B expression levels were found to be inversely correlated, whereas the overexpression of ATXN7L3B induces the decrease of ATXN7L3 expression levels and consequent loss of deubiquitinase activity (Li et al. 2016b). Other functional studies suggested that *ATXNL3B* behaves as a long noncoding RNA (*lnc-SCA7*) regulating *ATXN7* expression, an interaction that appears to be mediated by miR-124 micro-RNA through a negative feedback loop. It was proposed that polyQ-expanded ATXN7 reduces miR-124 transcription due to impairment of the SAGA complex, affecting the repression of the miRNA’s targets, *ATXN7* and *ATXN7L3B*, which will trigger cytotoxic nuclear inclusions primarily in the retina and the cerebellum (Tan et al. 2014).

ATXN7L3 and ATXN7L3B present lower protein conservation and different individual functions from ATXN7, while *ATXN7L1* and *ATXN7L2* appear to be more suitable for the functional compensation of the partial loss of the parental gene in SCA7 (Helmlinger et al. 2004; Lang et al. 2011). In fact, *ATXN7L1* and *ATXN7L2* paralogs resulted from the DNA-based duplication of *ATXN7* and take interchangeable roles in the SAGA’s DUBm, possibly being mutually exclusive, *i.e*., only one of these three homologs can be incorporated into a unique module (Vermeulen et al. 2010; Helmlinger and Tora 2017) (Fig. 2d). Moreover, both ATXN7 and ATXN7L2 incorporated in the STAGA’s DUBm complex appeared to be substituted by ATXN7L1 at later stages of the erythroid cells’ development (Papadopoulos et al. 2015). Still, there is a clear lack of studies exploring ATXN7 paralogs function in SAGA’s DUBm complex and transcriptional regulation, and little is known about their tissue expression.

#### SCA17: *TBP* gene

*TBP* (6q27) encodes for a widely expressed transcription initiation factor that integrates a larger complex along with TBP-associated factors (TAF1-15), constituting the core of the TFIID transcription preinitiation complex (Mishal and Luna-Arias 2022). In SCA17, the polyQ-expanded TBP impairs the DNA-binding and transactivation activity of the native protein and exacerbates the formation of neuronal nuclear inclusions, which reduce the TFIID complex availability for the transcription initiation process (Hsu et al. 2014; Yang et al. 2016) (Fig. 2e).

The *TBP* gene has two known paralogs, TBP like 1 (*TBPL1*, known as *TRF2*, *TLF*, *TLP*, or *TRP*) and 2 (*TBPL2*, also called *TBP2*/*TRF3*). *TBPL1* (6q23.2) resulted from a DNA-based duplication event of *TBP* within the same chromosome (Duttke et al. 2014) (Table 2). Despite some similarities observed between the two homologous proteins (C-terminal core; 41% homology), the domains ensuring the interaction with the TATA box do not seem to be preserved in TBPL1 (Rabenstein et al. 1999) (Fig. 3f). In fact, TBPL1 widely drives the transcription of TATA-less genes (*e.g.*, ribosomal protein genes) by relying on polypyrimidine initiator (TCT) motifs (Duttke et al. 2014) (Fig. 2e). Still, *TBPL1* seems to be differentially expressed with highest levels in testis resembling *TBP* (Ohbayashi et al. 1999b; Rabenstein et al. 1999). These homologous genes appear to regulate gene expression in a reciprocal and opposite manner (Rabenstein et al. 1999; Chong et al. 2005) and cannot replace each other’s functional properties (Moore et al. 1999; Teichmann et al. 1999; Dantonel et al. 2000; Kaltenbach et al. 2000). *TBPL1* and *TBP* seem to have evolved for different purposes, possibly regulating the transcription of different sets of genes and intervening at different stages of development (Rabenstein et al. 1999; Müller et al. 2001). One hypothesis is that the distinct N-terminus between the paralogs commit the transcription factors for specific regulatory signals or processes (Bondareva and Schmidt 2003). In fact, TBPL1 seems to be no longer needed for survival in more evolutionarily complex animals such as mice (impairment of spermiogenesis) (Martianov et al. 2001; Zhang et al. 2001), when compared to *C. elegans* (Dantonel et al. 2000; Kaltenbach et al. 2000) or *Xenopus laevis* (Veenstra et al. 2000; Jacobi et al. 2007) (defects in gastrulation and embryogenesis). In terms of transcriptional regulation, TBPL1 was considered a negative regulator of transcription, generally functioning as a signal-transducing transcription factor in cell cycle regulation and stress response (Dantonel et al. 2000; Shimada et al. 2003; Kieffer-Kwon et al. 2004; Chong et al. 2005; Tanaka et al. 2007), and directing the gene expression of target genes related to DNA replication and cell proliferation (Hochheimer et al. 2002; Park et al. 2006). Overall, *TBPL1* seems unlikely to substitute the parental gene functions as shown by *TBPL1* KO in mouse embryonic stem cells (Kwan et al. 2023).

*TBPL2* (14q22.3) probably resulted from a vertebrate-specific duplication event of *TBP*, being absent in lower eukaryotes, such as Drosophila and *C. elegans* (Persengiev et al. 2003; Bártfai et al. 2004) (Table 2). TBPL2 is a closely related TBP paralog with 95% identity in the C-terminal core (Persengiev et al. 2003; Akhtar and Veenstra 2011), able to bind to TATA promoters (Bártfai et al. 2004) (Figs. 2e, 3f). *TBPL2* was reported to be widely expressed in human tissues and cell lines as the parental gene, only showing some differences in the relative amount (Persengiev et al. 2003). However, it has become apparent that *TBPL2* is mainly expressed in oocytes and embryos (until the gastrula stage), showing an expression consistently lower than its paralogs (Persengiev et al. 2003; Bártfai et al. 2004; Jallow et al. 2004). Interestingly, TBPL1 and TBPL2 appear to be involved in a distinct processes in mice, being required to ensure adequate transcription during spermiogenesis and oogenesis, respectively (Martianov et al. 2001; Zhang et al. 2001; Gazdag et al. 2007, 2009). Similar to *TBPL1*, *TBPL2* appears to have evolved distinctively according to the species, being involved in oocyte maturation in mice (Müller et al. 2001; Gazdag et al. 2007, 2009) but required for embryonic development in *Xenopus laevis* and zebrafish (Bártfai et al. 2004; Jallow et al. 2004; Jacobi et al. 2007). In particular, TBPL2 was shown to mediate RNA polymerase II transcription during the meiosis of oocytes in mice, whereas continuously replacing the TBP during the oogenesis (Gazdag et al. 2007, 2009; Akhtar and Veenstra 2009) (Fig. 2e). *TBP* KO in mice seems lethal at the embryonic blastocyst stage (Martianov et al. 2002; Kwan et al. 2023). *TBPL2* KO mice were found to be sterile showing that the substitution of TBP by TBPL2 during the development of oocytes might be crucial for adequate transcription during folliculogenesis (Gazdag et al. 2009). However, the same was not observed in muscle differentiation (Malecova et al. 2016). *Xenopus laevis* studies also seem to support that TBPL2 can partially restore the transcription of TBP-dependent genes in the absence of TBP in embryonic development. Also, relatively few genes seem to depend on TBP in the embryo, suggesting specific and partially redundant functions (Jallow et al. 2004; Jacobi et al. 2007) that can be studied in SCA17 animal models (Cui et al. 2017).

#### DRPLA: *ATN1* gene

*ATN1* (12p13.31) encodes for the atrophin protein, a class of conserved transcriptional co-repressors playing a crucial role in nuclear receptor signalling pathways (Okamura-Oho et al. 1999; Wood et al. 2000; Feng et al. 2004; Zhang et al. 2006; Wang et al. 2008) (Fig. 2f). Also, ATN1 has been propose to interact with cytoskeletal and ubiquitin ligase proteins (Wood et al. 1998; Hou and Sibinga 2009). This gene is present in vertebrates and belongs to the atrophin family of proteins, along with the arginine-glutamic acid dipeptide repeats (*RERE*) gene and the Drosophila atrophin (*Atro*) [termed Grunge (*Gug*)] (Wang and Tsai 2008). The polyQ-expanded ATN1 was found to generate inclusions, both localized in the cytoplasm and nucleus of neuronal cells, which disrupts native protein–protein interactions (Nowak et al. 2023). Another feature of DRPLA was the ubiquitination, abnormal phosphorylation, and cleavage of polyQ-expanded ATN1 (Yazawa et al. 1999; Okamura-Oho et al. 2003; Suzuki et al. 2010) (Fig. 2f). Moreover, the polyQ-expanded ATN1 was suggested to cause a toxic gain of function by inhibiting CBP-dependent transcription, which is essential for neuronal development (Shimohata et al. 2000; Nucifora et al. 2001). Interestingly, *ATN1* KO mice were neurologically not affected but the selective knock-down of *ATN1* or histone demethylase *LSD1* (positively regulates ATN1 expression) in mice was sufficient to cause a premature differentiation and reduction of neural progenitor cells (mostly radial glial cells) (Zhang et al. 2014). Yet, increasing the expression of ATN1 rescued the deficit of neural progenitor cells, suggesting that ATN1 depletion may have consequences in brain development and a LSD1 inhibitor could function as a treatment option for adult-onset DRPLA (Zhang et al. 2014).

In vertebrates, there is only one known *ATN1* paralog previously mentioned as part of the atrophin family (Yanagisawa et al. 2000). The *RERE* gene (1p36.23) is thought to be involved in transcription regulation and/or DNA binding, showing the ability to recruit histone deacetylases and serve as a transcriptional corepressor (Zoltewicz et al. 2004; Wang et al. 2006, 2008; Plaster et al. 2007; Shen et al. 2007) (Table 2; Fig. 2f). The presence of a dipeptide repeat motif-containing arginine-glutamic acid (RE) at its C-terminus gave rise to the designation of *RERE*, but this gene is also referred to as atrophin 2* (ATN2)* or *ATN1L*. *RERE* is critical for both mouse and zebrafish development and survival (Zoltewicz et al. 2004; Asai et al. 2006; Plaster et al. 2007; Kim et al. 2013), especially in cerebellar Purkinje cells (Kim and Scott 2014), raising the assumption that *ATN1* probably originated from a DNA-based duplication event of *RERE*. This would also explain why *RERE* is distributed across metazoans and *ATN1* is restricted to vertebrates (Shen and Peterson 2009). In addition, the inability to distinguish *ATN1* KO and wild-type mice groups (Shen et al. 2007) further suggests that RERE and ATN1 functions are not equivalent on development. Nevertheless, the homologous genes seem to be post transcriptionally regulated by the same miRNAs (miR-429/miR-200b) (Karres et al. 2007), which may be a conserved mechanism to fine tune their expression and limit histone deacetylases activity [of particular interest in neurodegenerative diseases (Shukla and Tekwani 2020)].

Interestingly, *RERE* has the particularity to encode for two distinct transcripts: a long form (RERE-L, 1559 amino acids) restricted to the pancreas and testis; and a shorter ubiquitous form (RERE-S, 990 amino acids) expressed at higher levels in the cerebellum, testis, uterus, prostate, skeletal muscle and kidney, similar to *ATN1* (Onodera et al. 1995; Yanagisawa et al. 2000; Waerner et al. 2001; Shen et al. 2007). In addition, the presence of RERE transcripts has been predominantly reported to concentrate in the nucleus of cells, with residual levels in the cytoplasm (Yanagisawa et al. 2000; Waerner et al. 2001). Nonetheless, expression levels of *RERE* are generally lower when compared to *ATN1* (Yanagisawa et al. 2000). Contrasting with ATN1, RERE-L presents a high level of homology with the metastasis-associated family of proteins within its additional sequence of 569 amino acids (in comparison with RERE-S) and includes a total of four domains in the N-terminal [bromo-adjacent homology (BAH), EGL-27 and MTA1 homology 2 (ELM2), SWI3/ADA2/N-CoR/TFIII-B (SANT), and ZnF/GATA] mostly involved with proteins implicated in transcriptional regulation, followed by an atrophin 1 related domain (C-terminus region, 67% homology) (Yanagisawa et al. 2000; Bowen et al. 2004; Wang et al. 2006, 2008). RERE-S only contains the atrophin 1 domain, and is transcribed through an internal promoter (Shen et al. 2007) (Fig. 3g). RERE-S protein shares 50.8% identity with ATN1, suggesting highly conserved regions and overlapping functions (Shen and Peterson 2009). However, the atrophin domain generally has two structures: the conserved N-terminal, containing a nuclear localization signal, and a C-terminal interrupted by a nuclear export signal; reinforcing that atrophin-1 function is yet to be understood. Contrarily to ATN1, RERE has functional domains (SANT) that allow its concentration in the nucleus, where it forms nuclear speckles (Yanagisawa et al. 2000; Wang et al. 2006; Shen et al. 2007) and co-localizes with pro-apoptotic proteins (*e.g.*, PML and BAX) in promyelocytic leukemia oncogenic domains (Waerner et al. 2001) (Fig. 2f). Furthermore, RERE was suggested to control caspase-mediated cell death and cell survival (Waerner et al. 2001). In short, ATN1 resembles a truncated form of RERE, the portion of RERE that is missing from ATN1 could account for their functional differences while the conserved region could potentiate a functional overlap.

The previously mentioned RE repeat motifs are one common conserved regions between both homologs, though the precise function of this domain remains unclear (Yanagisawa et al. 2000; Shen and Peterson 2009). Nevertheless, RERE seems to directly interact with ATN1 partly via conserved RE motifs and form heterodimers, which can be related to the high tendency of this protein to aggregate with polyQ-expanded ATN1 (Yanagisawa et al. 2000). Therefore, the neuropathology occurring in DRPLA patients is likely to be accompanied by a depletion of the RERE protein due to this high affinity for the polyQ-expanded ATN1 aggregates. *RERE* heterozygous variants have been associated with neurodevelopmental disorder with or without anomalies of the brain, eye, or heart, with a loss of RERE function contributing to the development of orofacial clefts (Jordan et al. 2018; Kim et al. 2021). Besides, de novo variants in both *ATN1* and *RERE* seem to perturb the HX-repeat motif of the atrophin 1 domain (Fig. 3g), causing congenital anomalies such as hypotonia, epilepsy and developmental delay (Fregeau et al. 2016; Palmer et al. 2019), which further indicates a link between their functions.

## Conclusion and future perspectives

Seven SCAs are classified as polyQ diseases, as they result from the elongation of a glutamine tract, encoded by an expanded CAG repeat in the respective causative gene. So far, the pathological mechanisms underlying SCAs remain poorly understood, with just a few therapeutic approaches described to alleviate disease symptomatology. Cytotoxicity and neurodegeneration seem to occur primarily through a gain of function mechanism since polyQ expansions induce misfolding and aggregation inhibiting their typical interactions and/or promoting abnormal protein networks. Additionally, there has been increasing evidence for a partial loss of function effect responsible for disease-specific features as the biological activities of normal (non-expanded) proteins are partially lost and may be impaired by the polyQ-expanded form. Interestingly, some studies in SCA1 found that *ATXN1L*, a highly conserved paralog of the disease-associated *ATXN1* gene, was able to modulate the cytotoxicity of polyQ-expanded ATXN1 and suppress SCA1 symptomatology. The duplicate *ATXN1L* was suggested to compensate the ATXN1 native functions in the transcriptional ATXN1-CIC complex. Thus, an opportunity to further explore the etiology, pathological mechanisms and potential therapeutic targets may arise from the study of other SCA-associated paralogs that partially maintained the ancestral function of the respective parental genes over evolution. This may enrich the discussion around this group of SCA-associated genes, which has been studied over decades mostly under a very specific disease-oriented perspective.

Here, we reviewed the currently known similarities between polyQ SCA-associated genes and their human paralogs to ascertain functional redundancy, which would open an avenue to explore the hypothesis of duplicates being able to compensate for the partial loss of function reported in polyQ SCAs. The evolutionary relationships among paralogs may explain the degree of conservation between the parental gene and its duplicates, and elucidate about the potential functional redundancy of the homologous proteins. Future studies will certainly provide functional evidence that can grant us clues on common interactors and pathways regarding these paralogs. In this review, we guided the reader through the example of *ATXN1L* and its crucial role in SCA1, and next gathered data suggesting potential functional redundancy in additional seven duplicates of SCA-associated genes: *ATXN2L*, *ATXN3L*, *CACNA1B*, *ATXN7L1*, *ATXN7L2*, *TBPL2*, and *RERE*.

- ATXN2L is involved in stress granule and RNA metabolism, sharing highly similar protein domains and some common interactors with ATXN2, though little is known about its cellular function or association with SCA2 pathology.
- ATXN3L has been shown to analogously act as a deubiquitinase and is predicted to bind to the same interactors as ATXN3, suggesting a functional overlap that could restore the partial loss of function in MJD/SCA3. In addition, *ATXN3L* even presents a non-expanded (CAG)_n_ tract that opens a broad road to explore the evolution and expansion mechanisms underlying the CAG repeats at the parental *ATXN3*.
- CACNA1B is closely related to CACNA1A, presenting similar protein structures and synaptic functions, which suggests a high degree of functional overlap within Ca_v_2 channels that may be valuable in the context of SCA6.
- ATXN7L1 and ATXN7L2 have highly conserved domains and seem to play interchangeable roles with ATXN7 to ensure the adequate operation of the DUB module, as part of the SAGA complex. Nevertheless, there is a clear lack of studies exploring ATXN7 paralogs function in transcriptional regulation and involvement in SCA7 disease.
- TBPL2 seems to be the most promising paralog to compensate for TBP loss of function, since it contains a highly conserved core domain and common interactors with TBP (whereas TBPL1 does not bind to any TATA box-containing promoter). Still, further studies are needed to clarify the role of TBPL2 in transcription regulation and determine if a compensatory effect would be viable in SCA17.
- RERE belongs to the same family of proteins as the DRPLA causative protein, ATN1, playing similar roles in transcription repression. *ATN1* KO was not linked to any evident phenotype whereas RERE showed to be essential for both development and survival. These findings highly support the relevance of RERE in cellular function and corroborate the functional redundancy hypothesis that could be important in DRPLA.

In this review, we intended to draw attention to the study of duplicate genes in the context of polyQ SCA diseases, but certainly proteins with common domains may also be capable of functional redundancy. For instances, ATXN3 belongs to a family of deubiquitinating enzymes, which in addition to ATXN3L, includes JOSD1 and JOSD2, all sharing a highly conserved catalytic domain. Thus, all should be taken into consideration while exploring the molecular mechanisms of *ATXN3* and MJD pathogenesis. However, in functional studies of highly homologous proteins, one limitation researchers may face is the specificity of antibodies to distinguish proteins. The search for monoclonal customized antibodies with epitopes in less conserved regions of the proteins may help to overcome this concern.

In addition, bidirectional transcription of repeat expansions has been proposed to play a role in SCA pathogenesis. Hence, it would be interesting to gain insight on whether the presence of antisense genes for other parental/duplicated genes could affect expression levels of ataxin genes. Further studies on expression quantitative trait loci (eQTL) would probably explain variable levels of gene expression underlying these genes, ultimately elucidating some phenotypic variability observed in patients. The presence of possible haplotype effects (as well as interactions between variants, epistasis) could be considered to capture simultaneously the effect of multiple variants. Following these future research directions, it would be worthwhile exploring the potential of these genes that share functional activity to mitigate the effect of non-allele-specific therapeutic approaches on the normal alleles of polyQ SCA-associated genes. Treatments with small interfering RNAs, short hairpin RNAs, microRNAs, and antisense oligonucleotides have been performed in cell models and transgenic rodents in order to reduce exclusively mutant gene levels; however, when an allele-specific approach is not possible, duplicated genes may also be used as targets for therapeutic interventions.

### Supplementary Information

Below is the link to the electronic supplementary material.

Supplementary file1 (XLSX 13 KB)

## Data Availability

Data sharing does not apply to this article as no new data was generated or analysed for this review.
